# Pegfilgrastim-induced vasculitis of the subclavian and basilar artery complicated by subarachnoid hemorrhage in a breast cancer patient: a case report and review of the literature

**DOI:** 10.1186/s40792-022-01499-2

**Published:** 2022-08-12

**Authors:** Yukiko Seto, Nobuyoshi Kittaka, Azusa Taniguchi, Haruka Kanaoka, Satomi Nakajima, Yuri Oyama, Hiroki Kusama, Noriyuki Watanabe, Saki Matsui, Minako Nishio, Fumie Fujisawa, Koji Takano, Hideyuki Arita, Takahiro Nakayama

**Affiliations:** 1 Department of Breast and Endocrine Surgery, Osaka International Cancer Institute, 3-1-69 Otemae Chuo-ku, Osaka, 541-8567 Japan; 2 Department of Medical Oncology, Osaka International Cancer Institute, Osaka, Japan; 3 Department of Neurosurgery, Osaka International Cancer Institute, Osaka, Japan

**Keywords:** Breast cancer, Granulocyte-colony stimulating factor, Pegfilgrastim, Subarachnoid hemorrhage, Vasculitis

## Abstract

**Background:**

Pegfilgrastim (PEG) is a sustained-duration pegylated form of filgrastim, a granulocyte-colony stimulating factor agent that is widely used as prophylaxis against febrile neutropenia during chemotherapy. We report the case of a breast cancer patient who developed PEG-induced vasculitis complicated by subarachnoid hemorrhage (SAH) and review the relevant literature.

**Case presentation:**

A 48-year-old woman had undergone surgery for breast cancer and was receiving docetaxel and cyclophosphamide as adjuvant chemotherapy (docetaxel 75 mg/m^2^, cyclophosphamide 600 mg/m^2^); on day 4 of treatment, PEG had been administered. On day 14, she was admitted to hospital with fever, general malaise, and neck pain, and her C-reactive protein level was found to be high (12.65 mg/dL). Although infection was initially suspected, antimicrobial treatment was ineffective and other laboratory test results were negative for this. Contrast-enhanced computed tomography on day 22 showed thickened vessel walls in the left subclavian artery, the origin of the common carotid artery, and the thoracoabdominal aorta. On day 26, magnetic resonance imaging of the head to investigate possible causes of headache showed signs consistent with SAH, and magnetic resonance angiography images showed irregularity in the basilar artery wall; the findings of both studies were considered to be due to PEG-induced vasculitis. Once treatment with prednisolone 40 mg/day had started, the wall thickening and irregularity improved.

**Conclusion:**

Although an uncommon adverse effect, vasculitis affecting vessels of various sizes may be caused by PEG. To the best of our knowledge, this report is the first to describe a case of G-CSF-induced vasculitis complicated by SAH. In cases of persistent high fever and elevated inflammatory response after PEG administration and in the absence of infection, clinicians should consider the possibility of drug-induced vasculitis.

## Background

Pegfilgrastim (PEG) is a granulocyte-colony stimulating factor (G-CSF) with a sustained duration of action, which is due to the polyethylene glycol moiety attached to the N-terminal amino acid of filgrastim extending its half-life in the blood [1]. PEG is recommended for the prevention and management of febrile neutropenia (FN) during chemotherapy and is widely used in the treatment of various cancers. In the field of breast cancer, PEG is generally administered during adjuvant chemotherapy with dose-dense AC (doxorubicin plus cyclophosphamide) and TC (docetaxel plus cyclophosphamide) regimens.

Adverse events commonly associated with PEG include bone pain, back pain, and fever; however, these rarely lead to treatment discontinuation. Of greater concern is drug-induced vasculitis in blood vessels of various sizes, which is a known adverse effect of G-CSF agents; although their incidence is rare, capillary leak syndrome, cutaneous vasculitis, and large-vessel vasculitis have been reported [2].

According to a report by Oshima et al. in the Japanese Adverse Drug Event Report database, the frequency of G-CSF-induced large-vessel vasculitis in Japan is 0.47%, which is much higher than the 0.0014% reported for the USA [3]. Since 2015, the number of reported cases of G-CSF-induced vasculitis has been increasing, especially in Japan [4]. This may be partly due to increased awareness among clinicians, because in June 2018, the Japanese Ministry of Health, Labour and Welfare (MHLW) added large-vessel vasculitis to the list of serious adverse effects in the package insert for G-CSF preparations.

We report the case of a patient with PEG-induced vasculitis complicated by subarachnoid hemorrhage (SAH) and provide a review of the relevant literature. We also discuss the implications of the present case for clinical practice.

## Case presentation

A 48-year-old woman visited our hospital complaining chiefly of fever, general malaise, and left cervical pain. The patient had previously undergone unilateral mastectomy, axillary sentinel node biopsy, and axillary dissection. After surgery, she was taking loxoprofen, acetaminophen, mirogabalin, and mexiletine, in addition to using Chinese herbal medicine. She had started on TC (docetaxel 75 mg/m^2^, cyclophosphamide 600 mg/m^2^) as adjuvant chemotherapy. On day 4 of treatment, PEG had been administered for prevention of FN. On day 5, the patient had developed fever and arthralgia, and on day 7, a skin rash and pruritus had appeared on her neck and back. Although the symptom of itching had been relieved by treatment with an anti-allergic drug, she continued to have a fever of ≥ 38 °C and was admitted to hospital on day 14 for examination, investigations, and treatment.

On admission, the patient’s body height was recorded as 162 cm; body weight, 48.3 kg; body temperature, 37.6 °C; blood pressure, 91/47 mmHg; heart rate, 71 beats/min; and SpO_2_, 99% (in room air). She had clear consciousness, general malaise, and spontaneous pain and tenderness on the left side of the neck. There was no redness or swelling in the neck, and the cervical lymph nodes were not enlarged. Table 1 summarizes the laboratory values on admission; an elevated neutrophil-predominant white blood cell count of 14.11 × 103/μL and an elevated C-reactive protein (CRP) level of 12.65 mg/dL were observed, but procalcitonin level was normal and the results of blood and urine culture were negative. Plain computed tomography (CT) images of the chest and abdomen showed no obvious evidence of infection.

**Table 1:** Laboratory values on the patient’s admission to hospital

Variable	Laboratory value
Blood	
White blood cells, /μL	14.11 × 10^3^
Neutrophils, /μL	12.43 × 10^3^
Hemoglobin, g/dL	10
Platelets, /μL	172 × 10^3^
Biochemistry	
CRP, mg/dL	12.65
Procalcitonin, ng/mL	0.04
d-dimer, μg/mL	1.3
Total bilirubin, mg/dL	0.7
AST, U/L	36
ALT, U/L	25
Autoimmune antibodies	
MPO–ANCA, IU/mL	< 0.5
PR3–ANCA, IU/mL	< 0.5
C3, mg/dL	125
C4, mg/dL	27
Anti-DNA	< 2.0

*ALT* alanine aminotransferase, *AST* aspartate aminotransferase, *C3* complement 3, *C4* complement 4, *CRP* C-reactive protein, *DNA* deoxyribonucleic acid, *MPO–ANCA* myeloperoxidase–anti-neutrophil cytoplasmic antibody, *PR3–ANCA* serine proteinase 3–anti-neutrophil cytoplasmic antibody

**Fig. 1:**
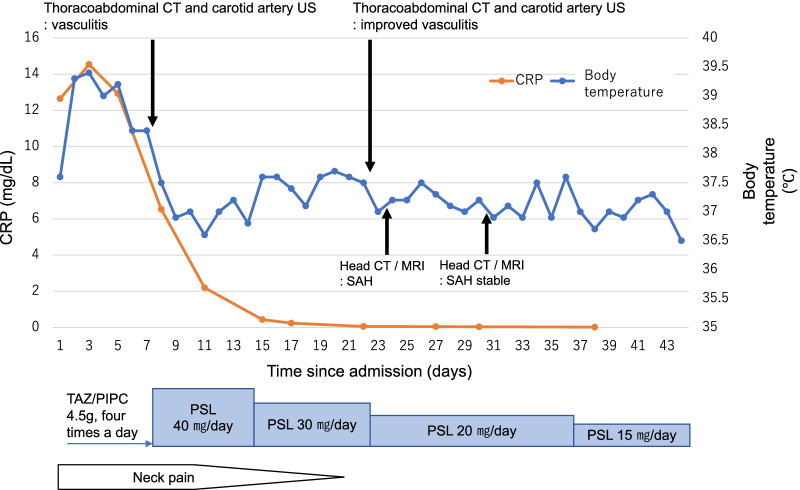
Disease course after admission. After admission, the patient received intravenous drip infusion of TAZ/PIPC; however, her fever, CRP level, and neck pain did not improve. On the 8th day of hospitalization, contrast-enhanced CT and cervical US showed vasculitis, and treatment with PSL 40 mg/day was started. On the 24th day, MRI of the head revealed SAH and vascular irregularity in the basilar artery. After contrast-enhanced CT and cervical US showed improvement in the vascular wall thickening, and the head MRI scan showed a trend toward improvement in the vascular irregularity, the patient was discharged on the 44th day. *CRP* C-reactive protein, *CT* computed tomography, *MRI* magnetic resonance imaging, *PSL* prednisolone, *SAH* subarachnoid hemorrhage, *TAZ/PIPC* tazobactam–piperacillin; *US* ultrasonography

Figure 1 shows the course of the patient’s condition after admission. Because of the possibility of infection, intravenous drip infusion of tazobactam–piperacillin (TAZ/PIPC 4.5 g, four times a day), a broad-spectrum antibacterial medication, was started on the day after admission. However, the patient continued to have fever ≥ 38 °C and left cervical pain, and right cervical pain newly appeared. Despite the administration of TAZ/PIPC, blood test results showed no reduction in inflammatory response, and therefore infection was considered unlikely to be the cause of the patient’s symptoms.

**Fig. 2:**
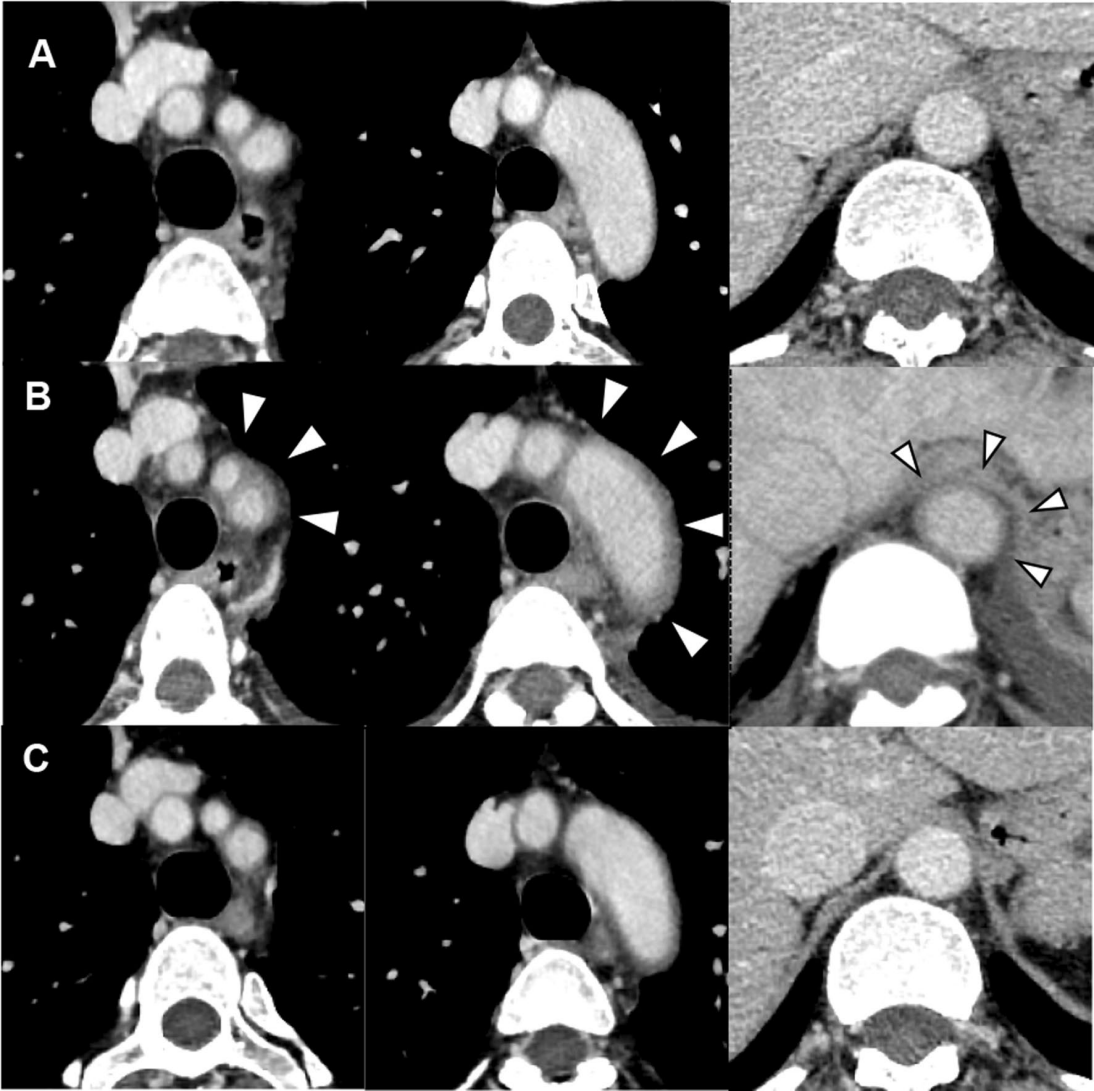
Contrast-enhanced CT images before and after treatment. **A** CT images before chemotherapy. **B** Contrast-enhanced CT images before treatment (day 8), showing thickened vessel walls in the left subclavian artery, the origin of the common carotid artery, and the thoracoabdominal aorta (white arrowheads). **C** Contrast CT images after treatment (day 22), showing improvement in the perivascular wall thickening. *CT* computed tomography

**Fig. 3:**
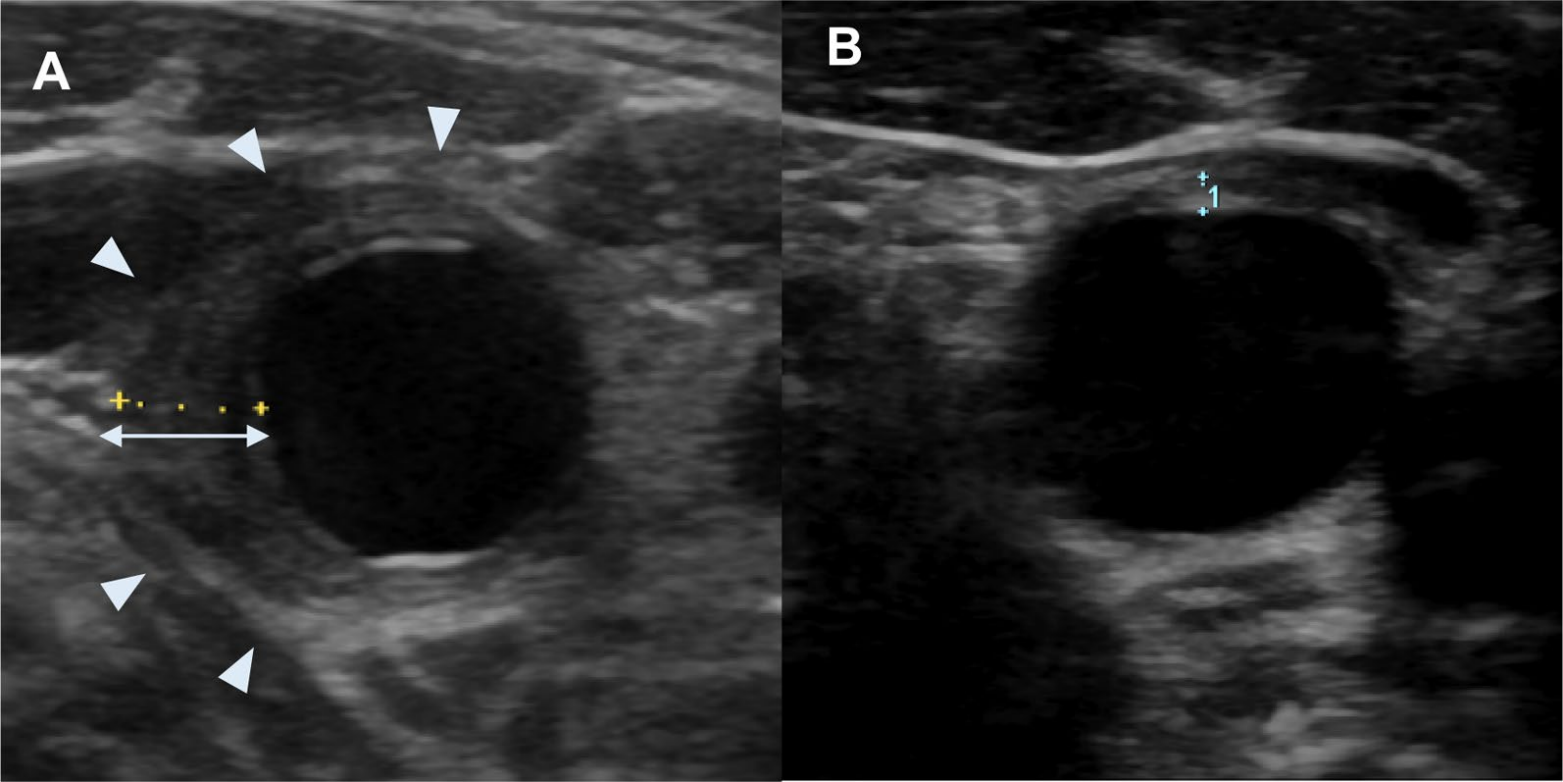
Carotid artery US images before and after treatment. **A** US image of the right internal carotid artery on day 8 of hospitalization, revealing hypoechoic or isoechoic wall thickening around the vessels from the bilateral common carotid bifurcation to the bilateral internal carotid arteries (white arrowheads). **B** US image of the right internal carotid artery on day 22 of hospitalization, showing improvement in the vessel wall thickening after treatment. *US* ultrasonography

On the 8th day after admission (day 22 of treatment), contrast-enhanced CT (Fig. 2) and carotid artery ultrasonography (Fig. 3) were performed to investigate the possibility of vasculitis, based on the persistent high fever and neck pain. The CT images showed thickened vessel walls in the left subclavian artery, the origin of the common carotid artery, and the thoracoabdominal aorta (Fig. 2B), suggesting vasculitis. The carotid artery ultrasonography revealed hypoechoic or isoechoic wall thickening around the vessels from the bilateral common carotid bifurcation to the bilateral internal carotid arteries (Fig. 3A), thus providing further evidence of vasculitis. Levels of myeloperoxidase–anti-neutrophil cytoplasmic antibody (MPO–ANCA), serine proteinase 3–anti-neutrophil cytoplasmic antibody (PR3–ANCA), and complements C3 and C4 were all normal, and the test result for anti-deoxyribonucleic acid (DNA) antibodies was negative, excluding the possibility of autoimmune disease.

Because the patient’s symptoms appeared immediately after PEG administration, we diagnosed PEG-induced vasculitis and started treatment with prednisolone (PSL) 40 mg/day on the same day. After administration of PSL, her fever rapidly resolved, CRP level decreased, and the cervical pain gradually eased until resolving completely by the 20th day of hospitalization (day 34). Images of the neck obtained by CT (Fig. 2C) and ultrasonography (Fig. 3B) on the 22nd day of hospitalization (day 36) showed clear improvement in the thickening of the vessel walls.

**Fig. 4:**
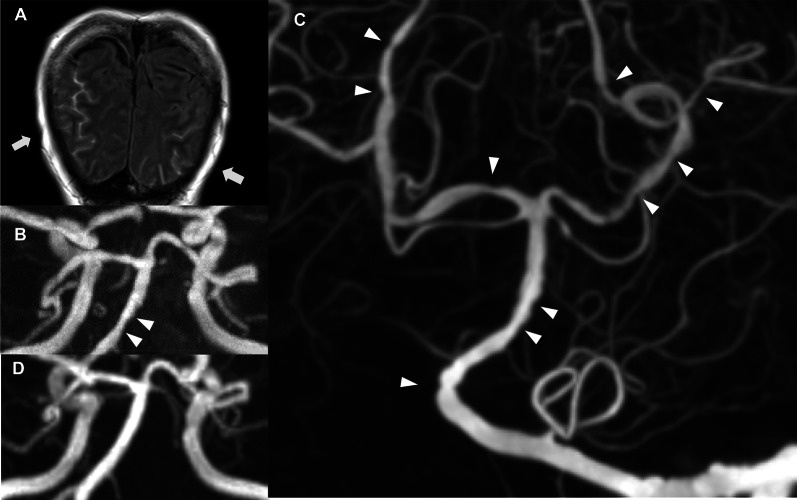
MRI images of the head.** A** Fluid-attenuated inversion recovery image on day 24 of hospitalization. The high-signal area along the sulcus suggested SAH (white arrows). **B** MRA image on day 24 of hospitalization, showing wall irregularity in the basilar artery (white arrowheads). **C** Cerebral angiography on day 26 of hospitalization. No signs of cerebral aneurysm were observed. Wall irregularities are visible in the left vertebral artery, basilar artery, and bilateral posterior cerebral arteries (white arrowheads). **D** MRA image 5 months after discharge. The irregularities in the wall of the basilar artery had completely disappeared. *MRA* magnetic resonance angiography, *MRI* magnetic resonance imaging, *SAH* subarachnoid hemorrhage

On the fourth day of hospitalization (day 18), the patient developed headache, but this fluctuated from day to day. On the 24th day of hospitalization (day 38), magnetic resonance imaging (MRI) of the head was carried out to investigate possible causes of the headache; this revealed high signal along the bilateral occipital sulci, as shown in the fluid-attenuated inversion recovery image (Fig. 4A). Additionally, images obtained by magnetic resonance angiography (MRA) showed irregularity in the wall of the basilar artery (Fig. 4B). Cerebral angiography performed on the 26th day of hospitalization (day 40) did not show any cerebral aneurysm (Fig. 4C), but wall irregularities were observed in the vertebrobasilar artery and bilateral posterior cerebral arteries. Based on these imaging results, we considered SAH associated with vasculitis to be the most probable cause of the patient’s headache.

After the patient had received follow-up treatment with supplemental fluid, images from MRI of the head carried out on the 31th and 38th day of hospitalization (days 45 and 52, respectively) showed no enlargement of the hemorrhage area, and MRA images confirmed that the vascular irregularities were improving. The patient was discharged on the 44th day after admission to hospital (day 58).

While the patient was followed up as an outpatient, the dose of PSL was gradually tapered until the treatment was discontinued, and the MRA image obtained at 5 months after discharge shows that the vascular irregularity has improved (Fig. 4D), with no sign of SAH recurrence. Since her discharge from the hospital, she has been receiving tamoxifen as adjuvant therapy, but TC therapy has not been resumed.

## Discussion

We have described a case of PEG-induced vasculitis of the subclavian and basilar artery complicated by SAH. To the best of our knowledge, this is the first report of a case of G-CSF-induced vasculitis complicated by SAH. The patient developed skin rash, fatigue, and neck pain after PEG administration; left cervical pain was her chief complaint during hospitalization. Although the skin rash and fatigue were likely to have been adverse effects of TC therapy, left cervical pain is not a typical adverse effect of TC. The patient reported, through a detailed interview, that her physical condition started to feel abnormal after taking PEG, which led us to strongly suspect the possibility of a PEG-induced adverse effect. Furthermore, the imaging findings excluded the possibility of infection or autoimmune disease as the cause, and no local skin changes or enlarged lymph nodes were observed. Consequently, we concluded that PEG-induced vasculitis was the most probable cause.

Table 2 summarizes details of the present case in addition to all 25 cases of vasculitis associated with PEG administration identified through our literature search for G-CSF-associated vasculitis, from the first, published in 2017, to the present. Although PEG was approved by the US Food and Drug Administration and by the European Medicines Agency in 2002, and has been marketed in Japan since 2014, it was not until 2017 that the first of these cases reports was published. Of the cases identified, 88% (23/26) of the patients were women, median age was 66 (43–76) years, and 58% (15/26) of patients had breast cancer as the primary disease. Median time to onset of symptoms after PEG administration was 8 (1–17) days; and most patients were hospitalized within 2 weeks of PEG administration. In 7 cases, including the present case, large-vessel vasculitis was observed not only in the aorta, but also in the common carotid artery, which is a major branch of the carotid artery.

**Table 2:** Previously reported cases of PEG-associated vasculitis

Reference	Year	Age (years)	Sex	Primary disease	Symptoms		CRP (mg/dL)	Time between PEG administration and symptoms (days)	Location of vasculitis	Diagnostic modality	Steroid
					Fever	Other					
Koyama et al. [19]	2021	43	F	Breast cancer	+		27.1	5	Arch/TA	CT	PSL 60 mg
Fujiwara et al. [20]	2021	66	F	Colon cancer	+	Back pain	20.2	2	Arch/TA, SCA	CT	PSL 30 mg
Kametani et al. [21]	2021	56	M	Mucinous chondrosarcoma	+	General fatigue, rash with pain	38.8	4	Arch/TA	CT	PSL 60 mg
Saito et al. [22]	2021	71	F	Intrahepatic cholangiocarcinoma	+	Back pain, chest pain	18.9	7	Arch/TA	CT	PSL 30 mg
Jimbo et al. [1]	2021	58	F	Breast cancer	+		13.7	8	SCA	CT	None
Lee et al. [7]	2020	66	F	Breast cancer	+	Myalgia, chills, nausea	26.6	13	Arch/TA, AA, CCA, innominate artery	CT	PSL 0.5 mg/kg
		49	F	Breast cancer	+	Myalgia, chest discomfort, dyspepsia	21.8	15	Arch/TA, CCA	CT, PET/CT	PSL 0.5 mg/kg
		50	F	Breast cancer	+	Myalgia, chills	29.6	12	Arch/TA, CCA, SCA, innominate artery	CT	PSL 0.5 mg/kg
		59	F	Breast cancer	+	Myalgia, chills	32.9	17	Arch/TA, AA, CCA, innominate artery	CT	PSL 0.5 mg/kg
		53	F	Breast cancer	+	Myalgia, chills, headache	10.9	14	Arch/TA, CCA	CT	PSL 0.5 mg/ kg
Nakamura et al. [23]	2020	66	F	Breast cancer	+	Left anterior neck pain	8.2	11	CCA	CT	None
Mukai et al. [24]	2020	66	F	Breast cancer	+	Malaise, abdominal discomfort	20.4	10	Arch/TA, AA	CT, MRI	PSL 55 mg
Taimen et al. [8]	2020	53	F	Breast cancer	+	Chest pain, sore throat, earache, dyspnea	Unknown	1	Arch/TA	CT, MRI	Corticosteroid
Miyazaki et al. [25]	2020	65	F	Pancreatic cancer	+			8	Arch/TA	CT	None
Hoshina et al. [26]	2019	72	F	Breast cancer	+		46.4	4	Arch/TA	PET/CT	None
Yukawa et al. [27]	2019	71	F	Endometrial cancer	+		27.9	4	Arch/TA	CT	PSL 60 mg
Sasaki et al. [28]	2019	69	M	Non-Hodgkin lymphoma	+		21.4	13	SCA	CT	None
Lardieri et al. [29]	2018	72	F	Uterine cancer		Cough, lumbar and back pain	30.1	13	Aorta	CT	None
		76	F	Breast cancer	+	Precordial pain	35.9	7	Aorta	CT	None
		61	F	Breast cancer	+	Left neck pain	25.4	7	Aorta	CT, MRI	None
		65	F	Breast cancer	+	Chest tightness, cough	30.8	9	Aorta	CT	Steroid
		66	M	Prostate cancer	+		33.3	8	Aorta	CT	None
		69	F	Esophageal cancer	+			11	Aorta	CT, US	None
Sato et al. [6]	2017	67	F	Lung cancer	+	Malaise	20.2	8	Arch/TA, CCA	CT, US	mPSL 80 mg
Present case	2022	48	F	Breast cancer	+	Malaise, left neck pain	12.65	14	Arch/TA, AA, CCA	CT, US	PSL 40 mg

*AA* abdominal aorta, *Arch* aortic arch, *CCA* common carotid artery, *CRP* C-reactive protein, *CT* computed tomography, *F* female, *M* male, *mPSL* methylprednisolone, *MRI* magnetic resonance imaging, *PEG* pegfilgrastim, *PET* positron emission tomography, *PSL* prednisolone, *SCA* subclavian artery, *TA* thoracic aorta, *US* ultrasonography

Most patients received corticosteroid treatment; prednisolone was administered at a dose of 0.5–1.0 mg/kg/day (absolute dose, 30–60 mg/day). In the present case, the patient also received prednisolone 40 mg/day. Symptoms were found to improve rapidly in all patients who received corticosteroid treatment. In addition to the case of SAH described in this report, G-CSF-associated vasculitis complicated by aneurysm [5] and aortic dissection [6] have also been reported, and thus, early diagnosis and therapeutic interventions, such as corticosteroid treatment for vasculitis, may be needed to prevent serious complications.

In a systematic review of data from 57 patients with G-CSF-induced vasculitis [4], 91% (52/57) were found to be women, median age was 60 (40–77) years, and 47% (27/57) had breast cancer as the primary disease. Although any G-CSF preparation can cause vasculitis, 67% (38/57) of the cases were reported to have been caused by a sustained-duration form of G-CSF. This is consistent with the trend observed in cases listed in Table 2, including the present case.

Regarding concomitant chemotherapy, in 40% of the identified cases of vasculitis (including the present case), the patients had been treated with a taxane-based regimen; however, other anticancer agents had been used in another 10% of cases. This suggests that vasculitis can be caused by G-CSF agents used in combination with any types of chemotherapy, although there may be synergic effects between taxane agents and G-CSF agents [7, 8]. Because vasculitis has been reported to have improved in all cases after restarting treatment with chemotherapy alone after discontinuing G-CSF, and there have been no reports of vasculitis caused by taxanes alone, we can conclude that G-CSF was the main cause of the patient’s condition, while perhaps also bearing in mind that vasculitis is especially likely to occur when G-CSF is used in combination with taxanes. G-CSF-induced vasculitis may tend to occur in middle-aged women with breast cancer, who are likely to be treated with a taxane-based regimen; however, we were unable to identify any specific risk factors through our literature search.

Although restarting G-CSF is not recommended, the TC regimen may be restarted in cases in which the vasculitis has fully resolved. However, in the present case, although the vasculitis had improved, a serious complication (i.e. SAH) had occurred, therefore we decided not to restart the TC regimen.

Although the mechanism underlying G-CSF-induced large-vessel vasculitis has not been fully clarified, it has been suggested to involve induction of immune mediators such as interleukin (IL)-2 and IL-6, leading to generation of pathological Th17 cells [6]. Sato et al. reported a case in which IL-6 was elevated at the onset of aortitis, but decreased during its improvement, suggesting that activation of antigen-specific CD4+ T cells, as stimulated by IL-6, may promote autoimmunity [6]. Furthermore, it has been suggested that phagocytosis and/or enzymatic activity resulting from activation of neutrophil precursors may be the cause of wall damage [5].

Takayasu arteritis (TAK) and giant cell arteritis (GCA) are diseases known to cause large-vessel vasculitis [9]. In TAK, vasculitic pain is experienced at the site of vasculitis, and stenosis and dilation of the vessels are observed on imaging. Both conditions have many similarities to G-CSF-induced vasculitis: elevated CPR in the blood test results, more frequent occurrence in middle-aged women, and higher prevalence in East Asia [3, 7]. As in G-CSF-induced vasculitis, inflammatory mediators (e.g. IL-6) and Th17 are known to be involved in the pathogenesis of TAK and GCA [10], and a similar genetic predisposition (e.g. Th17 pathway) may explain the high prevalence in East Asia. Moreover, there have been reports of G-CSF-induced vasculitis leading to aortic dissection [6], and of GCA being triggered by G-CSF administration [11], suggesting that G-CSF-induced large-vessel vasculitis may be caused by the same mechanism underlying TAK and GCA.

It has been reported that 15% of cases of non-traumatic SAH do not originate from an aneurysm (e.g. ruptured cerebral aneurysm), and that of these non-aneurysmal cases, two-thirds are due to perimesencephalic hemorrhage and one-third due to rare conditions including hemorrhage associated with inflammatory lesions of cerebral arteries, non-inflammatory lesions of intracerebral vessels, vascular lesions in the spinal cord, sickle cell disease, and the use of certain drugs [12]. In cases of inflammatory lesions of cerebral arteries, the possibilities of primary angiitis and angiitis secondary to autoimmune diseases (e.g. Behçet’s disease and polyarteritis nodosa) can be considered [13–15]. In the present case, the patient had no notable history of disease or injury other than breast cancer, and no cerebral aneurysm was detected on cerebral angiography, which together suggest that she had non-traumatic non-aneurysmal SAH. MRA images obtained several months after treatment for PEG-induced vasculitis showed improvement in the irregularities of the cerebral vessel wall, and no evidence of recurrence. Because the condition was reversible, the wall irregularities were unlikely to have been caused by underlying disease, so we considered it to be a case of G-CSF-induced vasculitis. Autoimmune vasculitis can be classified according to the size of the vessels affected (i.e. large-, medium- or small-vessel vasculitis) [16]. By contrast, G-CSF-induced vasculitis can cause vasculitis in vessels of any size [2].

Mechanisms suggested for development of non-aneurysmal SAH associated with vasculitis include small-vessel vasculitis as well as chronic hypoperfusion and hypoxia following cerebral vascular stenosis, leading to angiogenesis with formation of abnormal, and therefore rupture-prone, collateral vessels [17, 18]. In the present case, the localized nature of the SAH suggests that the hemorrhage originated from peripheral vessels of the posterior cerebral artery or from the neovascular vessels. In summary, PEG caused large-vessel vasculitis of the thoracoabdominal aorta and common carotid artery, which triggered small-vessel vasculitis in the brain, resulting in SAH as a complication.

## Conclusions

Vasculitis associated with PEG is under-recognized in clinical practice; however, cases involving cerebrovascular vessels can be fatal, so clinicians should be alert to this possibility. Drug-induced vasculitis should be considered in patients experiencing persistent fever and elevated inflammatory response after PEG administration, and when infection has been excluded as a cause. In such cases, early diagnosis and therapeutic intervention may prevent serious complications.

## Data Availability

Not applicable.

## Abbreviations

AA: Abdominal aorta
AC: Doxorubicin plus cyclophosphamide
ALT: Alanine aminotransferase
Arch: Aortic arch
AST: Aspartate aminotransferase
CCA: Common carotid artery
CT: Computed tomography
C3: Complement 3
C4: Complement 4
CRP: C-reactive protein
DNA: Deoxyribonucleic acid
F: Female
FN: Febrile neutropenia
G-CSF: Granulocyte-colony stimulating factor
GCA: Giant cell arteritis
IL: Interleukin
M: Male
MRA: Magnetic resonance angiography
MRI: Magnetic resonance imaging
MPO–ANCA: Myeloperoxidase–anti-neutrophil cytoplasmic antibody
PEG: Pegfilgrastim
PET: Positron emission tomography
PSL: Prednisolone
PR3–ANCA: Serine proteinase 3–anti-neutrophil cytoplasmic antibody
SAH: Subarachnoid hemorrhage
SCA: Subclavian artery
TA: Thoracic aorta
TAK: Takayasu arteritis
TAZ/PIPC: Tazobactam/piperacillin
TC: Docetaxel plus cyclophosphamide
US: Ultrasonography
